# Microbial Dynamics in Mixed-Culture Biofilms of *Salmonella* Typhimurium and *Escherichia coli* O157:H7 and Bacteria Surviving Sanitation of Conveyor Belts of Meat Processing Plants

**DOI:** 10.3390/microorganisms11020421

**Published:** 2023-02-07

**Authors:** Xianqin Yang, Hui Wang, Scott Hrycauk, Devin B. Holman, Tim C. Ells

**Affiliations:** 1 Agriculture and Agri-Food Canada Lacombe Research and Development Centre, Lacombe, AB T4L 1W1, Canada; 2 Agriculture and Agri-Food Canada Kentville Research and Development Centre, Kentville, NS B4N 5A7, Canada

**Keywords:** bacteria surviving sanitation, *E. coli* O157:H7, *Salmonella*, meat processing surfaces, biofilm, *Pseudomonas*

## Abstract

Biofilm formation can lead to the persistence of *Salmonella* Typhimurium (ST) and *E. coli* O157:H7 (O157). This study investigated the impact of meat processing surface bacteria (MPB) on biofilm formation by O157 (non-biofilm former; NF) and ST (strong biofilm former; BF). MPB were recovered from the contacting surfaces (CS), non-contacting surfaces (NCS), and roller surfaces (RS) of a beef plant conveyor belt after sanitation. O157 and ST were co-inoculated with MPB (CO), or after a delay of 48 h (IS), into biofilm reactors containing stainless steel coupons and incubated at 15 °C for up to 144 h. Coupons were withdrawn at various intervals and analyzed by conventional plating and 16S rRNA gene amplicon sequencing. The total bacterial counts in biofilms reached approximately 6.5 log CFU/cm^2^, regardless of MPB type or development mode. The mean counts for O157 and ST under equivalent conditions mostly did not differ (*p* > 0.05), except for the IS set at 50 h, where no O157 was recovered. O157 and ST were 1.6 ± 2.1% and 4.7 ± 5.0% (CO) and 1.1 ± 2.2% and 2.0 ± 2.8% (IS) of the final population. *Pseudomonas* dominated the MPB inocula and biofilms, regardless of MPB type or development mode. Whether or not a pathogen is deemed BF or NF in monoculture, its successful integration into complex multi-species biofilms ultimately depends on the presence of certain other residents within the biofilm.

## 1. Introduction

Shiga toxin-producing *Escherichia coli*, in particular the serotype O157:H7, is a significant foodborne human pathogen, and beef is the most commonly identified vehicle of transmission in *E. coli* O157:H7 outbreaks in North America [1]. Cattle are asymptomatic carriers of *E. coli* O157:H7 [2]. *Salmonella* can be found not only in the gastrointestinal tract of cattle, but also in other organs, such as lymph nodes, without causing overt disease in the host [3,4]. Thus, healthy cattle can carry these two pathogens, with the prevalence varying by animal source and rearing practices. Recurrence of the same strain at the same processing facility and its meat products over an extended period of time has been reported for *E. coli,* including *E. coli* O157:H7 [5,6,7]. This persistence has largely been attributed to the biofilm-forming ability of strains originating from the animals that can adapt to and become established in the meat processing environment [8,9]. Similarly, strong biofilm-forming *Salmonella* strains were among those which were recovered from beef trim [10].

Biofilms are surface-attached, organized microbial communities of aggregated cells, embedded in a hydrated matrix of self-produced extracellular polymeric substances (EPS) [11]. The most significant characteristic of biofilms in relation to the food processing environment is their increased resistance to sanitizing treatments compared to their planktonic counterparts. This resistance is mediated through the physical barrier provided by EPS, efflux systems, differentiation of bacterial cells into a dormant state, and the modification of the microenvironment, which can render a particular sanitizer less effective [12]. The daily cleaning and sanitization of processing equipment in meat processing facilities is a multi-step process, aiming to achieve two main objectives: visibly clean equipment (the removal of food residues that support microbial growth) and a reduction in the number of bacteria to acceptable levels [13]. Effective sanitation can reduce indicator organisms by up to 3 log units for food-contacting surfaces and 1 log for non-food contacting surfaces of conveyor belts [14]. Inadequately cleaned surfaces may promote soil build-up, in addition to having higher surviving microbial load. Regardless, the cleaning and sanitization program is not intended to achieve sterility for surfaces, and, as such, various bacterial species can be found on cleaned surfaces [14,15]. The attachment and subsequent biofilm formation of bacteria depend on interactions between three main components: the bacterial cells, the attachment surface, and the surrounding medium [16]. During operations, the wetting of conveyor belts by meat juice and the adsorption of food residues to surfaces provide a conditioning layer, which modifies surface properties favorably for bacterial attachment and subsequent growth.

The large body of information available on biofilm formation by *E. coli* O157:H7 and *Salmonella* in single-species cultures and, to a lesser extent in dual-species cultures, shows that biofilm formation by both species is highly strain-dependent and is impacted by companion species, with effects ranging from antagonistic to neutral to synergistic [10,17,18]. Impact of other bacteria on the biofilm formation of these pathogens has mainly been assessed in dual cultures using individual strains isolated from meat processing facilities. Published accounts on the interactions of the two pathogens with the equipment surface bacterial consortium surviving sanitation on the whole are largely lacking. In addition, it has been reported that the fate of a non-biofilm-forming *E. coli* strain can be altered by synergistic interactions and co-adhesion mechanisms with adherence-proficient bacteria in dual-species cultures [19]. Thus, a better understanding of the microbial dynamics in mixed biofilms by the bacterial population surviving sanitation, as well as *Salmonella* and *E. coli* O157:H7, and the ability of these two pathogens to insert into established mixed-species biofilms, could lead to the development of effective intervention strategies to control biofilms harboring these pathogens in the food processing environment. Therefore, the aim of the present work was to determine the dynamics of mixed-species cultures of post-sanitation equipment surface bacteria and *E. coli* O157:H7 and *S*. Typhimurium.

## 2. Materials and Methods

### 2.1. Bacterial Strains and Inoculum Preparation

Two pathogenic bacterial strains were included in the study. *E. coli* O157:H7 strain 1934 (O157), originally isolated from beef, was kindly provided by Dr. Alex Gill of Health Canada. *Salmonella enterica* serovar Typhimurium (ATCC 14028) (ST) was obtained from the American Type Culture Collection (ATCC). *E. coli* O157:H7 and *S.* Typhimurium were each cultured in 10 mL of Lennox broth (LB; BD Difco, Fisher scientific, Mississauga, ON, Canada), with the omission of salt (LB-NS), at 35 °C in a shaking incubator operated at 80 rpm for 16 h [20]. The cultures were transferred once, and the overnight cultures of the second transfer were further diluted in LB-NS to approximately 10^4^ CFU/mL. These cell suspensions were regarded as the pathogen inocula.

**Figure 1:**
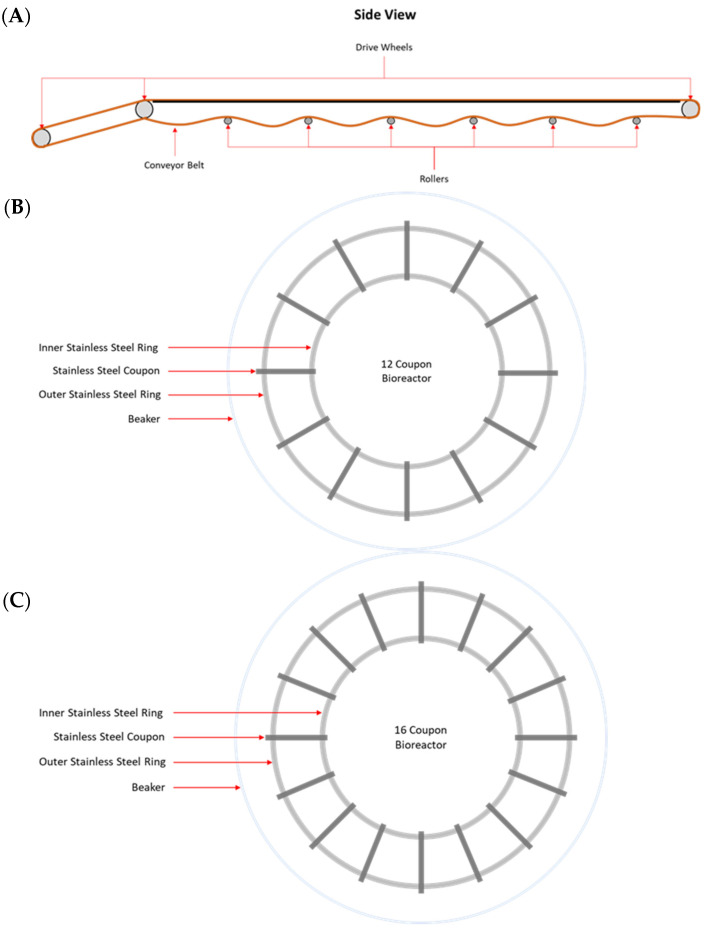
Schematic drawing of a side view of the rollers of a conveyor belt (**A**) and co-development (**B**) and insertion (**C**) bioreactors used in this study.

MPB were collected from the main conveyor belt on the fabrication floor of a federally inspected beef processing facility in Alberta, Canada. On each of three Wednesdays at bi-weekly intervals, samples were collected from the contacting surface (CS), non-contacting surface (NCS), and the surface of the roller (RS) mechanisms of the conveyor belt. The samples were collected by swabbing with sterile sponges (Speci-sponge; VWR Canlab, Mississauga, ON, Canada), each pre-moistened with 10 mL of 0.1% peptone water (*w*/*v*; BD Difco). For CS and NCS, a surface area of approximately 1000 cm^2^ was swabbed for each sample. Rollers are positioned approximately 180 cm apart along the conveyer belt and are 58 cm long, with a 4.5 cm outer diameter (Figure 1A). The total estimated area of each roller is 874.5 cm^2^, of which 550 cm^2^ was swabbed. For each surface type, three swab samples were collected at each sampling time. All sponge samples were placed on ice and processed within 1 h of sample collection.

An additional 10 mL of 0.1% peptone water was added to each stomacher bag containing a sponge, which was subsequently pummeled in a stomacher (Seward, Worthing, UK) at high setting for 2 min. The stomacher fluid from the three samples from the same surface type on the same sampling day was pooled, and the composite samples were regarded as MPB inocula, representing surface areas of 3000, 3000, and 1650 cm^2^ for CS, NCS, and RS, respectively. These MPB inocula were referred to as CS-MPB, NCS-MPB, and RS-MPB in this work. A 3 mL aliquot from each MPB inoculum was withdrawn for determination of initial bacterial counts.

### 2.2. Biofilm Development and Sampling

Biofilms were developed under static conditions in model in-house bioreactors, in which stainless steel coupons (20 × 25 × 4 mm) were held by two circular rings at the bottom of a 2 L beaker (Figure 1B,C). An aliquot of 10 mL from an MPB inoculum and 10 mL of each pathogen inoculum (~10^4^ CFU/mL) were added to a set of three bioreactors, each containing 970 mL of LB-NS and 12 stainless steel coupons (Figure 1B). These bioreactors were referred to as the co-development bioreactor set in this work. Another set of three bioreactors, each containing 970 mL of LB-NS and 16 stainless steel coupons (Figure 1C), were each inoculated with 10 mL of MPB inoculum only, and they were referred to as the insertion bioreactor set. All bioreactors were incubated at 15 °C for up to six days. The remaining stomacher fluid from each MPB inoculum was centrifuged at 11,000× *g* for 20 min at 4 °C, and the pellets were archived at −80 °C.

For the co-development set, 1.5 and 3 mL aliquots of the planktonic cultures, as well as four coupons, were withdrawn from each reactor at 2, 48, and 144 h. At each sampling time, the coupons were first withdrawn, and the planktonic cultures were then carefully mixed by pipetting up and down 20 times with a 25 mL serological pipet, without disturbing the coupons, prior to withdrawing aliquots of the cultures. The 1.5 mL planktonic culture aliquot withdrawn from each bioreactor was centrifuged at 11,000× *g* for 3 min at 4 °C, and the pellets were archived at −80 °C. For the insertion set, planktonic cultures and coupons were withdrawn at 2 and 48 h in the same manner as for the co-development set. To test the ability of the two pathogens to insert into existing biofilms, 10 mL of each pathogen inoculum was added to each of the insertion bioreactors after sampling at 48 h. The planktonic cultures and coupons were sampled at 50 h (2 h after the introduction of the pathogens) and 144 h in the same manner as before.

### 2.3. Enumeration of Bacteria by Plating

The 3 mL aliquot from each planktonic culture from the bioreactors was used for enumeration of bacteria. One ml of the original culture and appropriate dilutions in 0.1% peptone water were used to inoculate 3M Aerobic Count (AC) Petrifilm plates (3M, St. Paul, MN, USA) for determination of the total plate counts (TPC), following the manufacturer’s instructions. In addition, 100 µL of each original planktonic culture and appropriate dilutions were spread-plated on cefixime tellurite sorbitol (MacConkey) (CT-SMAC; Oxoid, Mississauga, ON, Canada) and xylose lysine deoxycholate (XLD; Oxoid) agars, for enumeration of *E. coli* O157:H7 and *Salmonella*, respectively. These plates were incubated at 35 °C for 24 h, and plates containing 30–300 colonies were used for enumerations. The 3 mL aliquot of each MPB inoculum was similarly processed for enumeration of the TPC.

The four stainless steel coupons removed from each bioreactor at each sampling time were processed, as described previously [20]. Briefly, loosely attached cells were removed by washing each coupon in succession in two 50-mL centrifuge tubes, each containing 15 mL of phosphate-buffered saline (pH 7.4) (PBS; Hardy Diagnostics, Santa Maria, CA, USA) for 1 min. Then, each washed coupon was transferred to a new 50-mL centrifuge tube containing 1.5 g of glass beads (500 µm; BioSpec Products, Bartlesville, OK, USA) and 15 mL of 0.1% peptone water. The attached cells were dislodged by vortexing for 1 min, and 12 mL was removed from each suspension. Suspensions from two coupons were used for determination of TPC, *E. coli* O157:H7, and *Salmonella* numbers, while the suspensions from the other two coupons were pelleted at 11,000× *g* for 20 min at 4 °C and archived −80 °C.

### 2.4. 16S rRNA Gene Amplicon Sequencing

In total, bacterial pellets from 198 samples were archived at −80 °C, including nine MPB inoculum samples, 63 planktonic cultures, and 126 biofilm samples. The estimated cell counts for 2 h insertion samples (n = 27) were ≤1 log CFU/mL and were therefore too low to be meaningful for 16S rRNA gene amplicon sequencing analysis. As such, they were excluded from further analysis. The remaining 171 samples were subjected to DNA extraction using a DNeasy Blood&Tissue kit (Qiagen, Toronto, ON, Canada), following the manufacturer’s instructions for extracting DNA from Gram-positive bacteria with slight modifications. The final elution of DNA was carried out with 100 µL sterile DNase and RNase free water, rather than elution buffer. Six negative extraction controls without any bacterial pellets were also included. The quality and quantity of the extracted DNA was determined using a Nanodrop spectrophotometer. The DNA was subjected to 16S rRNA gene amplicon library construction using primers targeting the V4 region of bacterial 16S rRNA gene [21]. The libraries were sequenced by Genome Quebec (Montreal, QC, Canada) using MiSeq (Illumina, Inc., San Diego, CA, USA) and the Illumina MiSeq Reagent Kit v2 (500 cycles). Of the 171 samples, 14 were removed due to low DNA quantity (Table S1).

### 2.5. 16S rRNA Gene Amplicon Analysis

The16S rRNA gene amplicon reads from the remaining 157 samples were processed using DADA2 1.14 in R (v. 3.6.3) with default parameters [22]. Primer sequences were removed using Cutadapt 2.10 [23] and subsequently trimmed to 120 (forward) and 180 bp (reverse), merged, and chimeras were removed using DADA2. Taxonomy was assigned to these amplicon sequence variants (ASVs) using the RDP classifier and the SILVA 16S rRNA gene database (release 138.1) [24]. No contaminants were identified using the “prevalence” method of the decontam R package v1.6.0 [25]. ASVs identified as mitochondria (n = 2) or chloroplasts (n = 5) were removed, along with any ASVs not assigned as bacteria (n = 1). One sample had only 60 reads, all belonging to one ASV, and it was removed from further analysis (Table S1). ASVs not assigned to a genus were manually BLASTed against the nr/nt database in GenBank. When the query ASV had ≥90% similarity with a sequence in GenBank, this taxonomy was assigned to that particular ASV. Otherwise, the ASVs were assigned to a higher taxonomic level.

### 2.6. Statistical Analysis

All bacterial counts were logarithmically transformed. The counts were converted to log_10_ CFU/mL and log_10_ CFU/cm^2^ for those from suspensions (planktonic cultures and inoculum) and coupons, respectively. The counts from coupons were regarded as viable bacterial counts for biofilms. The detection limits for the TPC, O157, and ST were 0, 1.0, and 1.0 log CFU/mL in suspensions, as well as 0.2, 1.2, and 1.2 log CFU/cm^2^ in biofilms, respectively. Bacterial counts were grouped according to bacterial type (TPC, O157, and ST), culture type (suspensions and biofilms), MPB type (CS, NCS, and RS), biofilm development type (co-development and insertion), and incubation time (2, 48, 50, and 144 h). When no CFUs were recovered from a sample for a bacterial type/group, a value of 0.5 log below the detection limit for that particular bacterial type/group was arbitrarily assigned for statistical analysis purposes. Two-way analysis of variance (ANOVA) for log counts and *post hoc* group comparisons using the Sidak test were performed in R.

For the 16S rRNA gene amplicon sequences, the ASV table produced by the DADA2 pipeline was further analyzed using phyloseq v. 1.30.0 [26] and vegan v. 2.5.2 [27] in R.

All samples were rarefied to 2725 reads, the lowest number of sequence reads among the 156 samples, so as to minimize the impact of uneven sequencing depths. Alpha diversity measures, including the number of observed ASVs and Shannon diversity and inverse Simpson diversity indices, were assessed using the Wilcoxon test for between group differences for MPB and 144 h samples. Beta diversity was assessed using the Bray-Curtis dissimilarities generated from rarified ASV tables. A principal coordinate analysis (PCoA) plot was used to visualize the Bray-Curtis dissimilarities. Permutational multivariate analysis of variance (PERMANOVA) using the ADONIS function with 10,000 permutations implemented in vegan was used to analyze the calculated Bray-Curtis dissimilarities.

A significance level of 0.05 was applied for all statistical analyses. All statistical analyses and graphing were performed in R (version 4.2.1).

## 3. Results

### 3.1. Bacterial Counts Determined by Plating

The TPC (mean ± STDEV) were 2.2 ± 1.4, 2.9 ± 1.1, and 3.4 ± 0.8 log CFU/mL for the CS-, NCS-, and RS-MPB inoculum, respectively, which corresponded to 3.2 ± 1.4, 3.9 ± 1.1, and 4.7 ± 0.8 log CFU/1000 cm^2^ for the respective surfaces. The numbers varied between the three trials, with the largest variation observed for CS-MPB counts, ranging from 0.7 (trial 1) to 3.3 (trial 3) log CFU/mL. The initial cell density for the O157 and ST overnight cultures was 8.5 ± 0.3 and 8.1 ± 0.4 log CFU/mL, respectively.

**Figure 2:**
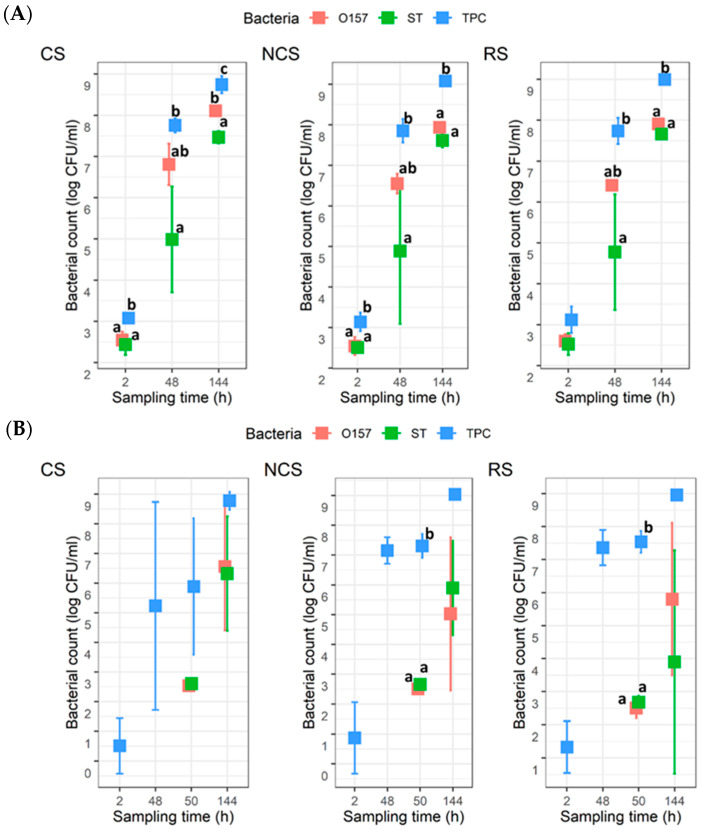
Total plate count (TPC), *Escherichia coli* O157:H7 (O157), and *Salmonella* Typhimurium (ST) counts in planktonic cultures (n = 3). Mixed cultures of the two pathogens and meat processing surface bacteria (MPB) in Lennox broth without salt were incubated at 15 °C for six days. O157 and ST were introduced simultaneously with MPB (**A**) or delayed by 48 h (**B**). MPBs were from the contact surfaces (CS), non-contact surfaces (NCS), and roller surfaces (RS) of the main conveyor belt on the fabrication floor of a beef processing facility. ANOVA was performed to compare the bacterial counts at each sampling time followed by a Sidak *post hoc* pairwise comparison. Significant (*p* < 0.05) differences are indicated by different lowercase letters.

For the co-development planktonic cultures, the TPC were around 3 log CFU/mL at 2 h, and they increased to approximately 9 log CFU/mL by 144 h for all three MPB types (Figure 2A). The counts for O157 and ST were approximately 2.5 log CFU/mL at 2 h, which then increased to 8.0 and 7.5 log CFU/mL, respectively, at 144 h. There were no statistical differences (*p* > 0.05) between the counts for O157 and ST at any given time for a given MPB type, except for CS at 144 h, where the mean count for O157 was ~0.7 log units higher (*p* < 0.05) than that for ST. For the insertion planktonic cultures, the mean TPC at 2 h were >0.5 < 1.3 log CFU/mL (Figure 2B), with the CS- and NCS-MPB each having one trial, which did not yield any TPC. Neither O157, nor ST, were recovered at 2 or 48 h. The numbers for the two pathogens at 50 h or 144 h were not significantly different (*p* > 0.05), regardless of MPB type. The mean TPC also reached approximately 9 log CFU/mL by 144 h, but it was different from the co-development planktonic cultures in that counts were not statistically different from those determined for either pathogen. It is noteworthy that there was large variability observed for the ST counts at 48 h with the co-development cultures and both pathogen counts at 144 h with the insertion cultures.

**Figure 3:**
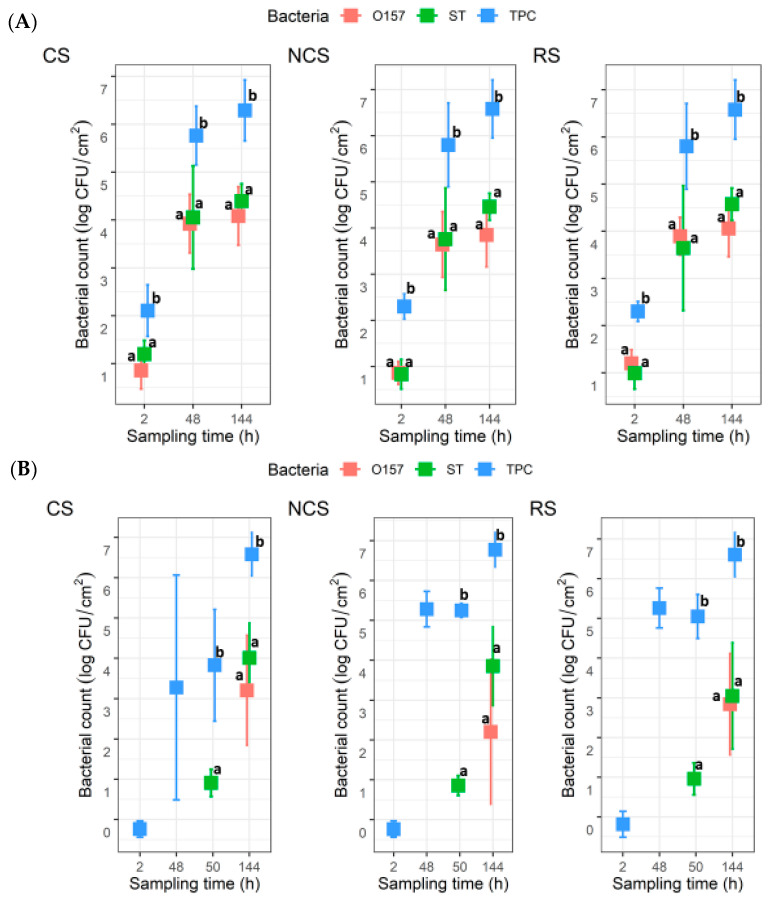
Total plate count (TPC), *Escherichia coli* O157:H7 (O157), and *Salmonella* Typhimurium (ST) counts in biofilms on stainless steel coupons (n = 6). The coupons were incubated at 15 °C for six days in Lennox broth without salt inoculated with O157, ST, and meat processing surface bacteria (MPB). O157 and ST were introduced with MPB (**A**) or delayed by 48 h (**B**). MPBs were from the contact surfaces (CS), non-contact surfaces (NCS), and roller surfaces (RS) of the main conveyor belt on the fabrication floor of a beef processing facility. ANOVA was performed to compare the bacterial counts at each sampling time, followed by a Sidak *post hoc* pairwise comparison. Significant (*p* < 0.05) differences are indicated by different lowercase letters.

For the co-development biofilms, all coupons (n = 6) for each MPB type at 2 h yielded TPC, with mean values of 2.1 ± 0.5, 2.3 ± 0.3, and 2.3 ± 0.2 log CFU/cm^2^ for CS-, NCS-, and RS-MPB, respectively (Figure 3A). For O157 and ST, however, the recovery from the six coupons from each of the three MPB was sporadic (one, two, and five coupons for O157; and five, one, and three coupons for ST). The mean counts for these two pathogens ranged from 0.8 to 1.2 log CFU/cm^2^. All three types of bacterial counts in biofilms increased (*p* < 0.05) with incubation time by 48 h, with final mean counts at 144 h, reaching ~6.5 log CFU/cm^2^ for TPC, 3.85 to 4.08 log CFU/cm^2^ for O157, and 4.4 to 4.6 log CFU/cm^2^ for ST across the three MPB types. No significant differences (*p* > 0.05) between counts for the two pathogens under equivalent conditions were observed, but both were significantly lower (*p* < 0.05) than the TPC. For the insertion set, only one of the six coupons from each MPB type at 2 h yielded TPC. Large variations (from no recovery to 5.6 log CFU/cm^2^) in TPC were observed for CS-MPB for up to 50 h. Nevertheless, mean TPC in biofilms increased to >6.5 log CFU/cm^2^ by 144 h (Figure 3B). It is noteworthy that no O157 were recovered from any of the coupons at 50 h, irrespective of MPB type, whereas the mean ST counts were approximately 1 log CFU/cm^2^. At 144 h, the numbers of the two pathogens did not differ significantly (*p* > 0.05). However, the numbers of both pathogens were significantly lower (*p* < 0.05) than the TPC, irrespective of MPB type, mostly by 2–3 log units.

For both planktonic cultures and biofilms, none of the three bacterial groups were significantly (*p* > 0.05) impacted by MPB type or the development mode. Additionally, no significant interaction effects between MPB type and incubation time were observed for bacterial counts in biofilms, except for TPC values of insertion biofilms (*p* < 0.05). However, there were no significant differences (*p* > 0.05) in these counts between the three MPB types at equivalent incubation times.

**Figure 4:**
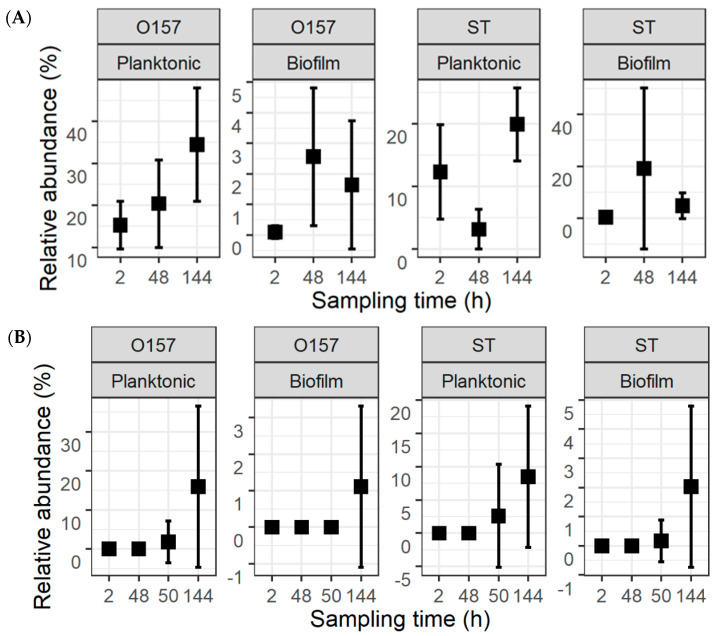
Relative abundance of *Escherichia coli* O157:H7 (O157) and *Salmonella* Typhimurium (ST) within the total population of bacteria. The pathogens were introduced simultaneously with meat processing surface bacteria (**A**) or delayed by 48 h (**B**). This is replotted with information in Figure 2 and Figure 3 to highlight the fractions of the two pathogens.

The fractions of the pathogens in the total population were calculated from their counts and the TPC (Figure 4). For the co-development cultures, the mean relative abundance values for O157 were 15.3 ± 5.7%, 20.4 ± 10.4%, and 34.5 ± 13.5%, in planktonic cultures, and 0.1 ± 0.2%, 2.6 ± 2.3%, and 1.6 ± 2.1%, in biofilms, at 2, 48, and 144 h, respectively. The respective numbers of ST in planktonic cultures and biofilms at these same time intervals were 12.3 ± 7.6%, 3.2 ± 3.2%, and 19.9 ± 5.9%, as well as 0.3 ± 0.7%, 19.1 ± 31.2%, and 4.7 ± 5.0%. For the insertion cultures, the mean fractions of O157 were 1.8 ± 5.4% and 15.9 ± 20.7% in planktonic cultures at 50 and 144 h, as well as 1.1 ± 2.2% in biofilms at 144 h. For ST, the mean fractions were 2.6 ± 7.6% and 8.5 ± 10.6% in planktonic cultures and 0.2 ± 0.7% and 2.0 ± 2.8% in biofilms at 50 and 144 h.

### 3.2. Microbial Composition, Diversity, and Structure, as Determined by 16S rRNA Gene Amplicon Sequence Analysis

Alpha diversity measures of the MPB inocula microbiota and the culture microbiota at 144 h were assessed. There were no significant differences (*p* > 0.05) for any of the alpha diversity measures between the three MPB inocula, between co-development and insertion for either biofilm or planktonic cultures from the same MPB, or between the planktonic cultures of the three MPB. For the biofilms, the CS-MPB had significantly lower (*p* < 0.01) alpha diversity than the NCS- and RS-MPB under insertion, as determined by both inverse Simpson and Shannon diversity indices, but not under co-development mode (*p* > 0.05).

**Figure 5:**
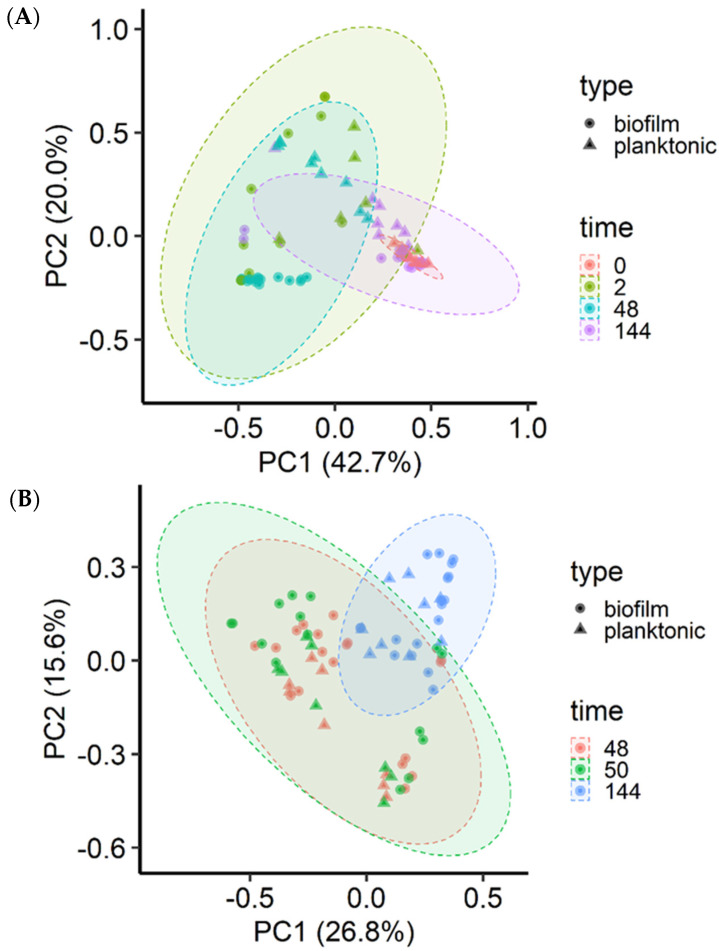
Principal coordinate analysis of the Bray-Curtis dissimilarities of the biofilm and planktonic microbiota. Meat processing surface bacteria (MPB), Salmonella Typhimurium, and *Escherichia coli* O157:H7 in Lennox broth without salt were incubated in biofilm reactors at 15 °C for six days and sampled at various time points (h). *S*. *Typhimurium* and *E*. *coli* O157:H7 were introduced simultaneously with MPB (**A**) or delayed by 48 h (**B**).

The microbial community structure was significantly (*p* < 0.001) affected by incubation time (R^2^ = 0.15), MPB type (R^2^ = 0.08), and culture type (planktonic vs. sessile; R^2^ = 0.05) for the insertion set, and only by incubation time (R^2^ = 0.20) and MPB type (R^2^ = 0.10) for the co-development set. The three MPB inocula did not differ significantly (*p* > 0.05) based on community structure. However, the final microbial communities at 144 h were significantly different from each other when grouped by MPB type (*p* < 0.001; R^2^ = 0.17), culture type (*p* = 0.01; R^2^ = 0.05), and mode of development (*p* = 0.04; R^2^ = 0.04). For the co-development set, most biofilm samples at 48 h clustered away from their corresponding planktonic cultures, and the MPB inocula and 144 h samples were tightly clustered (Figure 5A). For the insertion set, the clustering was diagonal to the axis (Figure 5B).

**Figure 6:**
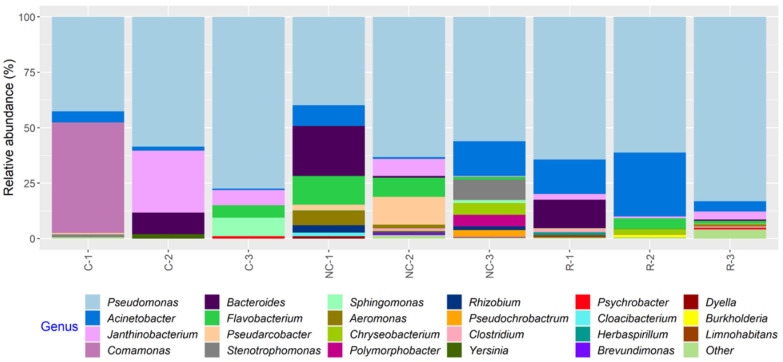
Relative abundance of bacterial genera in samples collected from equipment surfaces determined by 16S rRNA gene amplicon sequence analysis. The samples were collected on three days from the contact (C), non-contact (NC), and roller (R) surfaces of a conveyor belt in a beef processing facility. Genera with a relative abundance <1% in any of the samples were classified as “Other”.

In total, 23 bacterial genera appearing at a relative abundance ≥1% were found in the microbiota of the MPB inocula, and, in general, a greater number of taxa were found in samples from NCS and RS than CS (Figure 6; Table S2). Variations in the relative abundance of bacterial genera in samples from the same surface on different days and in samples from different surfaces on the same sampling day were observed. However, the relative abundance of *Pseudomonas* ranged from 39.2 to 83.1% and was the highest in all but C-1, in which *Comamonas* was at 49.9% (Table S2). Other relatively abundant genera (≥5% in one or more samples) found on all three surfaces included *Bacteroides*, *Janthinobacterium*, *Pseudarcobacter*, *Acinetobacter*, *Flavobacterium*, and *Aeromonas*.

**Figure 7:**
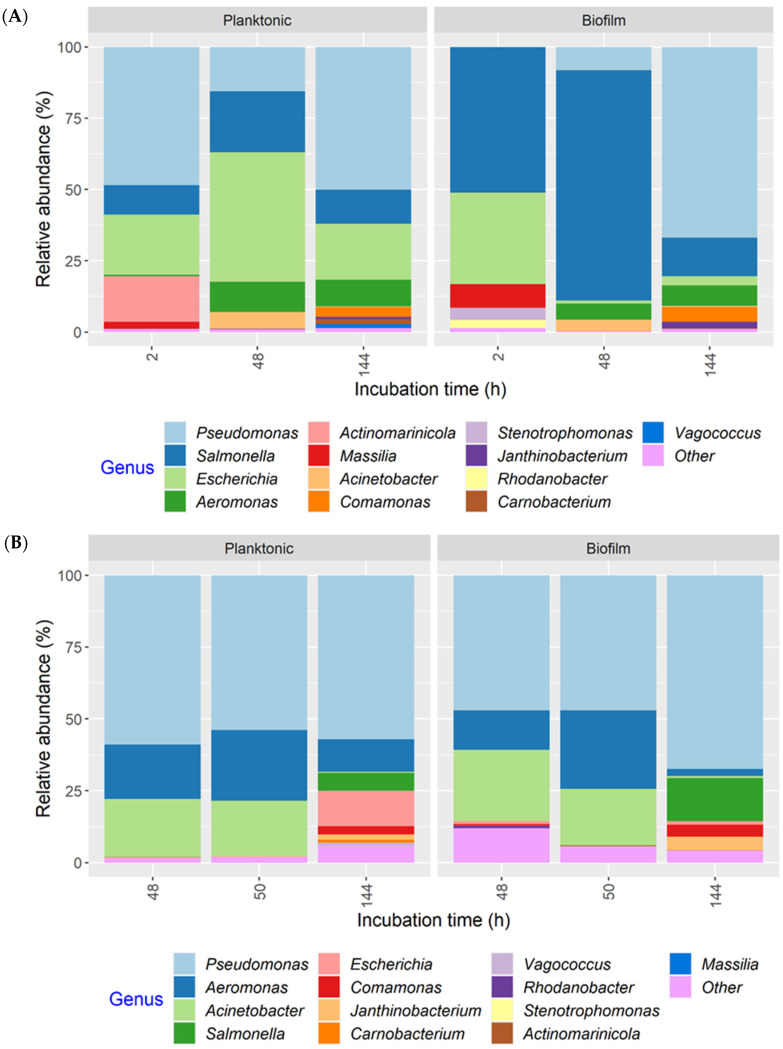
Relative abundance of bacterial genera in planktonic cultures and biofilms, as determined by 16S rRNA gene amplicon sequence analysis. The multispecies cultures were developed with meat processing surface bacteria (MPB), *Salmonella* Typhimurium, and *Escherichia coli* O157:H7 at 15 °C for six days, with the pathogens introduced simultaneously with MPB (**A**) or delayed by 48 h (**B**). Genera with a relative abundance <1% in any of the samples were classified as “Other”.

Regardless of the MPB or culture type, or development mode, *Pseudomonas* had the highest relative abundance in all cultures at the end of incubation (144 h), accounting for more than 50% of 16S rRNA gene sequences in most samples and close to 90% in some samples (Figure S1). Thus, the MPB types were pooled to illustrate population dynamics as a function of post-sanitation surface bacteria (Figure 7). The same 13 genera were found in both co-development and insertion sets. For co-development biofilms, *Salmonella* and *Escherichia* accounted for the majority of the population at 2 h (51.0 and 32.0%, respectively). At 48 h, *Salmonella* maintained its predominance, with a relative abundance of 81.0% compared to *Escherichia,* which was at only 1.0%. *Pseudomonas* increased in relative abundance from <1% at 2 h to 67.0% at 144 h. The mean relative abundance values of *Salmonella* and *Escherichia* at the end of incubation were 14.0% and 3.0%, respectively. Of note, *Massilia,* which was below 1% in the inocula, was found at 8.0 and 3.0% in 2 h biofilms and planktonic cultures, respectively, but was not detected at 48 or 144 h. Interestingly, strong initial, but weakened biofilm formation, was observed for *Massilia* in single-species biofilms, as well [28]. The co-development planktonic cultures differed from their biofilm counterparts in that *Escherichia* had a much higher relative abundance of 45.0 and 20.0% at 48 and 144 h, respectively, and *Pseudomonas* had a higher initial relative abundance (48.0%). For the insertion cultures, *Pseudomonas*, *Aeromonas,* and *Acinetobacter* were the three most relatively abundant genera at 48 and 50 h, collectively accounting for 85.0 and 93.0% of the total in biofilms and 97.0 and 95.0% in planktonic cultures. The relative abundance of both *Salmonella* and *Escherichia* was <1% under equivalent conditions when present. At 144 h, however, *Salmonella* and *Escherichia* increased to 15.0 and 1.0% in biofilms, as well as to 6.0 and 12.0% in planktonic cultures, respectively, largely at the expense of *Acinetobacter*. The remaining seven genera were all ≤5% at any given time.

## 4. Discussion

The attachment of a bacterial cell to a surface, the first step of biofilm formation, is dictated by its ability to overcome the repulsive force of the surface [16]. The surface charge and appendages used for attachment play an important role in that regard and vary between species and even among different strains. In addition, environmental factors can alter the surface properties for a particular bacterium, including the presence of companion species. Thus, the population structure of mixed-species biofilms is the net outcome of the microbial composition, as well as the competitiveness of individual members in terms of surface attachment, their resistance to antimicrobial compounds produced by other residents in the population [29], and growth rate under the given conditions. In this work, we investigated the microbial dynamics of mixed-species biofilms containing bacteria that survived the routine cleaning and sanitation of a conveyor belt in a beef processing plant, along with a non-biofilm-forming *E. coli* O157:H7 strain [18] and a strong biofilm-forming *S*. Typhimurium strain [28].

In the present work, we identified 23 bacterial genera with a relative abundance ≥1% in the microbiota of MPB inocula, which was recovered from conveyor belt surfaces, and the dominant genus was found to be *Pseudomonas.* The other genera with notably high relative abundance included *Comamonas*, *Acinetobacter*, *Flavobacterium*, *Pseudarcobacter*, *Bacteroides*, *Janthinobacterium*, and *Aeromonas*. Four genera (*Pseudomonas*, *Comamonas*, *Acinetobacter*, and *Flavobacterium*) were also found on surfaces after sanitation in a previous study, where the bacterial community at the same processing facility was examined during cleaning of the conveyor belts [14]. In that study, *Janthinobacterium* and *Aeromonas* were found before cleaning started. In the present study, some minor constituents that were found among the MPB in the inocula or during growth were also found in the previous study. The large overlap in bacterial genera detected at the same facility over a six-year period may suggest the presence of a stable core resident microbiota within the plant itself. A study, which examined the microbiota of conveyor belts in a salmon processing facility post-sanitation, reported that *Pseudomonas* was the most prevalent and abundant genus, followed by *Acinetobacter*, both of which were found in more than 50% of samples [15]. Brightwell et al. [30] reported that 84% of the bacterial isolates recovered from cleaned and sanitized conveyor belts in a lamb boning room were also *Pseudomonas* spp. The other relatively abundant bacteria in both studies were mostly Gram-negative. The bacterial genera identified on post-sanitation conveyor belts in the present study were in agreement with those previously reported from protein processing facilities, where operating temperatures are low and humidity is high [31].

Six relatively abundant (≥1%) bacterial genera were detected in biofilms at 144 h for both co-development and insertion sets. Similarly, the number of bacterial genera found in naturally occurring biofilms in a meat processing plant ranged from four (conveyor belt) to 12 (drain), a comparatively small number considering the diversity of bacteria naturally present in such environments [32]. *Pseudomonas* dominated mixed-species biofilms formed from a cocktail of 14 bacterial isolates that survived sanitation of conveyor belts in salmon processing plants [15]. *Pseudomonas* spp. were also among the most frequently isolated bacteria in biofilms in a meat processing facility where pork, poultry, and beef were processed during operation and after sanitation [33]. Biofilms on food contact surfaces in two meat plants that processed ham, meatballs, and sausages mainly consisted of *Pseudomonas* and *Acinetobacter* [34]. The predominance of *Pseudomonas* spp. in the present study was not observed in our previous work, where mixed-species biofilms were developed using a cocktail of 41 MPB strains in equal proportions together with the same O157 or ST strains included here [20]. This difference could have resulted from differences in both the composition of the MPB and/or the relative abundance of *Pseudomonas* in the initial inoculum, since its apparent higher levels in the present study may have given it a competitive advantage.

The introduction of O157 and ST affected the microbial dynamics in biofilms formed by MPB, especially during the early development stages. In the insertion cultures incubated for 48 h, the microbial composition of planktonic cultures and biofilms were largely similar and mainly consisted of three genera: *Pseudomonas*, *Aeromonas,* and *Acinetobacter*. The co-development biofilms at 2 h were dominated by ST and O157, even though *Pseudomonas* accounted for 50% of the total population in planktonic cultures. However, both pathogens were gradually displaced by *Pseudomonas* in co-development biofilms. This may suggest that the O157 and ST strains were able to adhere to the stainless steel surface more efficiently compared to the MPB, but they were less competitive when growing in biofilms under the conditions investigated. In meat plants, the air temperature in fabrication facilities where carcasses are fabricated to cuts and trimmings is maintained at 6–7 °C. Both *Salmonella* and *E. coli* are mesophilic organisms, and their growth at or below the operating temperature is limited [35]. During cleaning, the ambient temperature could rise to 15 °C [14]. Even so, the more psychrotrophic *Pseudomonas*, *Aeromonas,* and *Acinetobacter* may have a competitive edge at these temperatures. Compared to when they were initially introduced or six days after introduction, more variation was observed in the counts for the two pathogens at 48 h for ST in the co-development set and 144 h for both in the insertion set of planktonic cultures and in biofilms. Differences in the ability/opportunity to attach to different coupons/surfaces in the same bioreactor or in different trials may have contributed to this variability for biofilms. This variation may also be attributed to differences in the initial cell density and microbial population and the permissibility that affected the growth of O157 and ST. It is noteworthy that the relative abundance of both O157 and ST, as determined by 16S rRNA gene amplicon sequencing, was not always congruous with the culturing data. This discrepancy could have been caused by differences in DNA extraction efficiency and PCR amplification bias for different bacterial species [36].

For the co-development cultures at 2 h, both ST and O157 were among the major species in the total microbial population (>10% as determined by plating), with no difference (*p* > 0.05) in their counts at this time or at any given time. These findings are in agreement with a previous study, which found that the two pathogens co-established in mixed-species biofilms, comparably, when each was co-inoculated individually with a mock community of 41 MPB strains [20]. The surface colonization of an *E. coli* O157:H7 strain that could not form biofilms on its own was greatly enhanced when co-cultured with an *Acinetobacter calcoaceticus* isolate recovered from a meat processing environment after disinfection [37]. A generic *E. coli* strain, PHL565, was not able to adhere to a glass surface on its own, but it could do so when co-cultured with *Pseudomonas putida* MT2 [19]. Here, biofilm formation in mixed-species cultures by the otherwise curli- and cellulose-deficient non-biofilm-forming O157 strain [18] was likely aided by the presence of companion strains, to a level comparable to the strong biofilm-forming ST strain. However, O157 did not insert into established biofilms as efficiently as ST, as reflected by the lack of recovery of O157 from any of the coupons from the insertion cultures at 50 h. Even so, the numbers of O157 and ST in insertion biofilms did not differ significantly by 144 h, regardless of surface type.

In conclusion, the findings of this work demonstrated that Gram-negative bacteria were prevalent among the equipment post-sanitation microbial community, with *Pseudomonas* being most predominant, and *Comamonas*, *Acinetobacter*, *Flavobacterium*, *Janthinobacterium*, and *Aeromonas* were represented in sizable fractions. Despite the difference in biofilm-forming ability of the O157 strain and the ST strain in single-species cultures, they mostly did not differ in both mature co-development and insertion biofilms developed at temperatures relevant to meat processing facilities. Further work on gene expression levels would help to pinpoint the nature of the interactions in the mixed-species cultures. Nevertheless, the findings in this work underline the importance of considering the entire microbial community when developing strategies to control biofilms, rather than focusing on biofilm formation by individual strains/species.

## Data Availability

The sequencing data obtained in this study were deposited in NCBI under BioProject PRJNA894215. The accession numbers for 16S rRNA gene amplicon sequence read archive (SRA) are from SRR22058084 to SRR22058239.

## Supplementary Materials

The following supporting information can be downloaded at: https://www.mdpi.com/article/10.3390/microorganisms11020421/s1, Figure S1: Relative abundance of bacterial genera in planktonic cultures and biofilms of meat processing surface bacteria (MPB), *Salmonella* Typhimurium and *Escherichia coli* O157:H7. pathogens.; Table S1: Samples removed from analysis; Table S2: Bacterial genera composition in meat processing surface bacteria (MPB) inocula microbiota from contacting (C), non-contacting (NC), and roller (R) surfaces.

## References

[1] World Health Organization & Food, Agriculture Organization of the United Nations (2018). Shiga Toxin-Producing Escherichia coli (STEC) and Food: Attribution, Characterization, and Monitoring: Report.

[2] Pruimboom-Brees I.M., Morgan T.W., Ackermann M.R., Nystrom E.D., Samuel J.E., Cornick N.A., Moon H.W. (2000). Cattle lack vascular receptors for Escherichia coli O157:H7 Shiga toxins. Proc. Natl. Acad. Sci. USA.

[3] Nickelson K.J., Taylor T.M., Griffin D.B., Savell J.W., Gehring K.B., Arnold A.N. (2019). Assessment of Salmonella prevalence in lymph nodes of U.S. and Mexican cattle presented for slaughter in Texas. J. Food Prot..

[4] Webb H.E., Brichta-Harhay D.M., Brashears M.M., Nightingale K.K., Arthur T.M., Bosilevac J.M., Kalchayanand N., Schmidt J.W., Wang R., Granier S.A. (2017). Salmonella in peripheral lymph nodes of healthy cattle at slaughter. Front. Microbiol..

[5] Yang X., He A., Badoni M., Tran F., Wang H. (2017). Mapping sources of contamination of Escherichia coli on beef in the fabrication facility of a commercial beef packing plant. Food Control.

[6] Visvalingam J., Wang H., Youssef M.K., Devos J., Gill C.O., Yang X. (2016). Spatial and temporal distribution of Escherichia coli on beef trimmings obtained from a beef packing plant. J. Food Prot..

[7] Arthur T.M., Bono J.L., Kalchayanand N. (2014). Characterization of Escherichia coli O157:H7 strains from contaminated raw beef trim during “High Event Periods”. Appl. Environ. Microbiol..

[8] Wang R., Kalchayanand N., King D.A., Luedtke B.E., Bosilevac J.M., Arthur T.M. (2014). Biofilm formation and sanitizer resistance of Escherichia coli O157:H7 strains isolated from “high event period” meat contamination. J. Food Prot..

[9] Yang X., Wang H., He A., Tran F. (2018). Biofilm formation and susceptibility to biocides of recurring and transient Escherichia coli isolated from meat fabrication equipment. Food Control.

[10] Wang R., Schmidt J.W., Harhay D.M., Bosilevac J.M., King D.A., Arthur T.M. (2017). Biofilm formation, antimicrobial resistance, and sanitizer tolerance of Salmonella enterica strains isolated from beef trim. Foodborne Pathog. Dis..

[11] Hall-Stoodley L., Costerton J.W., Stoodley P. (2004). Bacterial biofilms: From the Natural environment to infectious diseases. Nat. Rev. Microbiol..

[12] Giaouris E., Heir E., Hébraud M., Chorianopoulos N., Langsrud S., Møretrø T., Habimana O., Desvaux M., Renier S., Nychas G.-J. (2014). Attachment and biofilm formation by foodborne bacteria in meat processing environments: Causes, implications, role of bacterial interactions and control by alternative novel methods. Meat Sci..

[13] Heinz G., Hautzinger P. Meat Processing Technology for Small- to Medium-Scale Producers. http://www.fao.org/docrep/010/ai407e/ai407e00.htm.

[14] Wang H., He A., Yang X. (2018). Dynamics of microflora on conveyor belts in a beef fabrication facility during sanitation. Food Control.

[15] Langsrud S., Moen B., Møretrø T., Løype M., Heir E. (2016). Microbial dynamics in mixed culture biofilms of bacteria surviving sanitation of conveyor belts in salmon-processing plants. J. Appl. Microbiol..

[16] Garrett T.R., Bhakoo M., Zhang Z. (2008). Bacterial adhesion and biofilms on surfaces. Prog. Nat. Sci..

[17] Wang R., Bono J.L., Kalchayanand N., Shackelford S., Harhay D.M. (2012). Biofilm formation by Shiga toxin-producing Escherichia coli O157:H7 and non-O157 strains and their tolerance to sanitizers commonly used in the food processing environment. J. Food Prot..

[18] Visvalingam J., Yang X. (2018). Inter- and intra-generic interaction between meat plant environmental bacteria and Escherichia coli O157:H7 in co-culture biofilms. International Association for Food Protection.

[19] Castonguay M.H., van der Schaaf S., Koester W., Krooneman J., van der Meer W., Harmsen H., Landini P. (2006). Biofilm formation by Escherichia coli is stimulated by synergistic interactions and co-adhesion mechanisms with adherence-proficient bacteria. Res. Microbiol..

[20] Visvalingam J., Wang H., Ells T.C., Yang X. (2019). Facultative anaerobes shape multispecies biofilms composed of meat processing surface bacteria and Escherichia coli O157:H7 or Salmonella enterica Serovar Typhimurium. Appl. Environ. Microbiol..

[21] Caporaso J.G., Lauber C.L., Walters W.A., Berg-Lyons D., Huntley J., Fierer N., Owens S.M., Betley J., Fraser L., Bauer M. (2012). Ultra-high-throughput microbial community analysis on the Illumina HiSeq and MiSeq platforms. ISME J..

[22] Callahan B.J., McMurdie P.J., Rosen M.J., Han A.W., Johnson A.J.A., Holmes S.P. (2016). DADA2: High-resolution sample inference from Illumina amplicon data. Nat. Methods.

[23] Martin M. (2011). Cutadapt removes adapter sequences from high-throughput sequencing reads. EMBnet J..

[24] Quast C., Pruesse E., Yilmaz P., Gerken J., Schweer T., Yarza P., Peplies J., Glöckner F.O. (2013). The SILVA ribosomal RNA gene database project: Improved data processing and web-based tools. Nucleic Acids Res..

[25] Davis N.M., Proctor D.M., Holmes S.P., Relman D.A., Callahan B.J. (2018). Simple statistical identification and removal of contaminant sequences in marker-gene and metagenomics data. Microbiome.

[26] McMurdie P.J., Holmes S. (2013). phyloseq: An R package for reproducible interactive analysis and graphics of microbiome census data. PLoS ONE.

[27] Oksanen J., Blanchet F.G., Friendly M., Kindt R., Legendre P., McGlinn D., Minchin P.R., O’Hara R.B., Simpson G.L., Solymos P. (2019). Vegan: Community Ecology Package.

[28] Visvalingam J., Zhang P., Ells T.C., Yang X. (2019). Dynamics of biofilm formation by Salmonella Typhimurium and beef processing plant bacteria in mono- and dual-species cultures. Microb. Ecol..

[29] Olejnik-Schmidt A.K., Schmidt M.T., Sip A., Szablewski T., Grajek W. (2014). Expression of bacteriocin divercin AS7 in Escherichia coli and its functional analysis. Ann. Microbiol..

[30] Brightwell G., Boerema J., Mills J., Mowat E., Pulford D. (2006). Identifying the bacterial community on the surface of Intralox belting in a meat boning room by culture-dependent and culture-independent 16S rDNA sequence analysis. Int. J. Food Microbiol..

[31] Fagerlund A., Langsrud S., Møretrø T. (2021). Microbial diversity and ecology of biofilms in food industry environments associated with Listeria monocytogenes persistence. Curr. Opin. Food.

[32] Belk A.D., Frazier A.N., Fuerniss L.K., Delmore R., Belk K., Borlee B., Geornaras I., Martin J.N., Metcalf J.L. (2022). A pilot study: The development of a facility-associated microbiome and its association with the presence of Listeria spp in one small meat processing facility. Microbiol. Spectr..

[33] Wagner E.M., Pracser N., Thalguter S., Fischel K., Rammer N., Pospíšilová L., Alispahic M., Wagner M., Rychli K. (2020). Identification of biofilm hotspots in a meat processing environment: Detection of spoilage bacteria in multi-species biofilms. Int. J. Food Microbiol..

[34] Caraballo Guzmán A., González Hurtado M.I., Cuesta-Astroz Y., Torres G. (2020). Metagenomic characterization of bacterial biofilm in four food processing plants in Colombia. Braz. J. Microbiol..

[35] EFSA BIOHAZ Panel (2014). Scientific Opinion on the public health risks related to the maintenance of the cold chain during storage and transport of meat. Part 1 (meat of domestic ungulates). EFSA J..

[36] Jovel J., Patterson J., Wang W., Hotte N., O’Keefe S., Mitchel T., Perry T., Kao D., Mason A.L., Madsen K.L. (2016). Characterization of the gut microbiome using 16S or shotgun metagenomics. Front. Microbiol..

[37] Habimana O., Heir E., Langsrud S., Asli A.W., Møretrø T. (2010). Enhanced surface colonization by Escherichia coli O157:H7 in biofilms formed by an Acinetobacter calcoaceticus isolate from meat-processing environments. Appl. Environ. Microbiol..

