# Supplementary material for: Microbial Dynamics in Mixed-Culture Biofilms of Salmonella Typhimurium and Escherichia coli O157:H7 and Bacteria Surviving Sanitation of Conveyor Belts of Meat Processing Plants
Source: Microorganisms. 2023 Feb 7;11(2):421. doi: 10.3390/microorganisms11020421 (PMC9960345; doi:10.3390/microorganisms11020421)
Supplement: Supplementary file 1 [file microorganisms-11-00421-s001.zip › Figure S1.pdf]

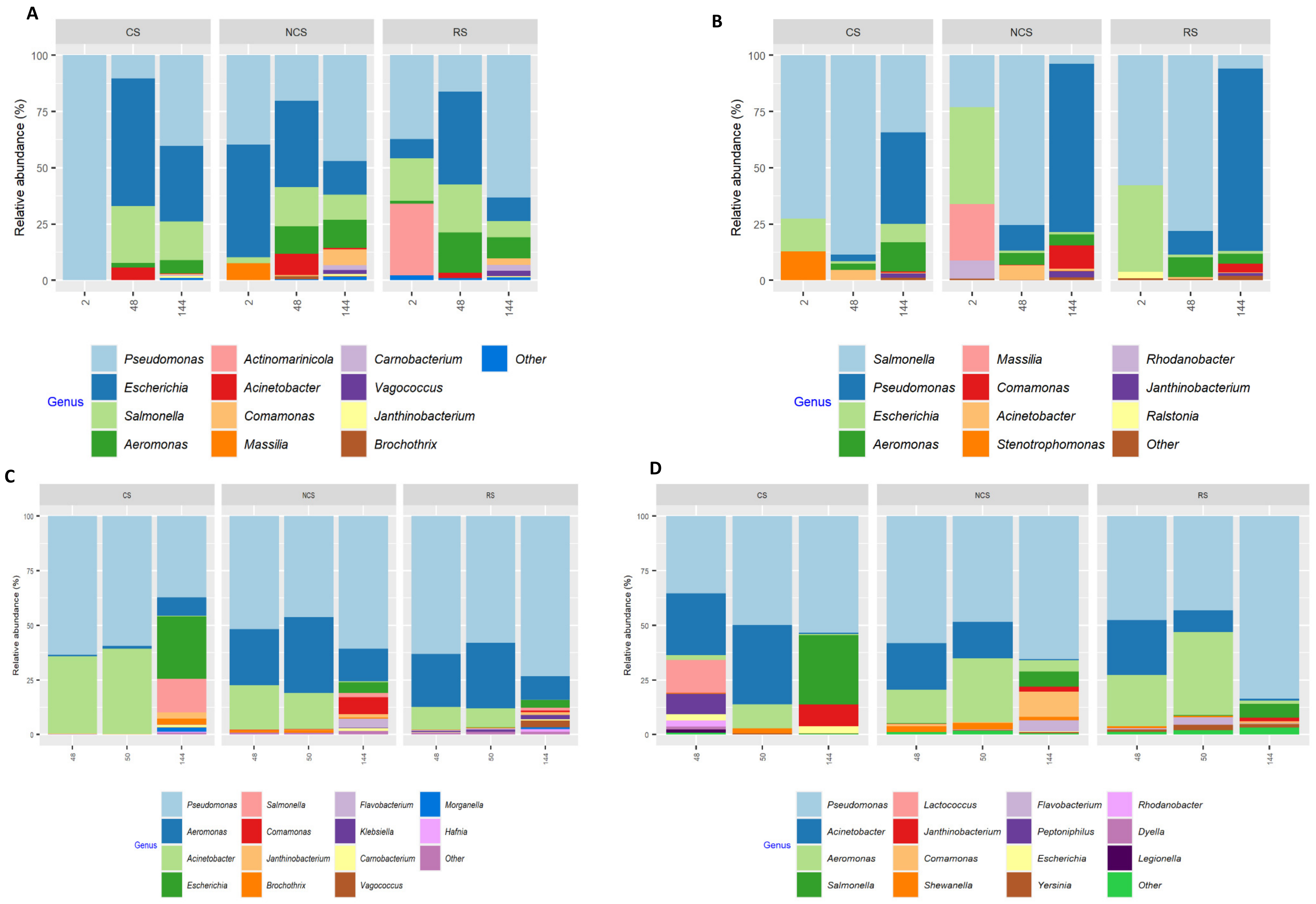

Figure S1. Relative abundance of bacterial genera in planktonic cultures and biofilms of meat processing surface bacteria (MPB), *Salmonella* Typhimurium and *Escherichia coli* O157:H7. *S*. Typhimurium and *E. coli* O157:H7 were introduced simultaneously with MPB (A, planktonic; B, biofilms) or delayed by 48 h (C, planktonic; D, biofilms) and incubated in biofilm reactors at 15°C for 6 days. MPB were collected from the contact (C), non-contact (NC) and roller (R) surfaces of a conveyor belt in a beef processing facility.
